# Analysis of Endocrine Disruption in Southern California Coastal Fish Using an Aquatic Multispecies Microarray

**DOI:** 10.1289/ehp.11627

**Published:** 2008-08-28

**Authors:** Michael E. Baker, Barbara Ruggeri, L. James Sprague, Colleen Eckhardt-Ludka, Jennifer Lapira, Ivan Wick, Laura Soverchia, Massimo Ubaldi, Alberta Maria Polzonetti-Magni, Doris Vidal-Dorsch, Steven Bay, Joseph R. Gully, Jesus A. Reyes, Kevin M. Kelley, Daniel Schlenk, Ellen C. Breen, Roman Šášik, Gary Hardiman

**Affiliations:** 1 Department of Medicine; 2 BioMedical Genomics Facility, School of Medicine, University of California, San Diego, La Jolla, California, USA; 3 Department of Experimental Medicine and Public Health, University of Camerino, Camerino, Italy; 4 Southern California Coastal Water Research Project, Costa Mesa, California, USA; 5 Los Angeles County Sanitation Districts, Whittier, California, USA; 6 Environmental Endocrinology Laboratory, California State University, Long Beach, California, USA; 7 Department of Environmental Sciences, University of California, Riverside, California, USA; 8 Division of Physiology; 9 Moore’s Cancer Center, University of California, San Diego, La Jolla, California, USA

**Keywords:** *Danio rerio*, endocrine disruptors, flatfish, hornyhead turbot, microarray, multispecies array, nonylphenol, *Pleuronichthys verticalis*, xenobiotics, xenoestrogens, zebrafish

## Abstract

**Background:**

Endocrine disruptors include plasticizers, pesticides, detergents, and pharmaceuticals. Turbot and other flatfish are used to characterize the presence of chemicals in the marine environment. Unfortunately, there are relatively few genes of turbot and other flatfish in GenBank, which limits the use of molecular tools such as microarrays and quantitative reverse-transcriptase polymerase chain reaction (qRT-PCR) to study disruption of endocrine responses in sentinel fish captured by regulatory agencies.

**Objectives:**

We fabricated a multigene cross-species microarray as a diagnostic tool to screen the effects of environmental chemicals in fish, for which there is minimal genomic information. The array included genes that are involved in the actions of adrenal and sex steroids, thyroid hormone, and xenobiotic responses. This microarray will provide a sensitive tool for screening for the presence of chemicals with adverse effects on endocrine responses in coastal fish species.

**Methods:**

We used a custom multispecies microarray to study gene expression in wild hornyhead turbot (*Pleuronichthys verticalis*) collected from polluted and clean coastal waters and in laboratory male zebrafish (*Danio rerio*) after exposure to estradiol and 4-nonylphenol. We measured gene-specific expression in turbot liver by qRT-PCR and correlated it to microarray data.

**Results:**

Microarray and qRT-PCR analyses of livers from turbot collected from polluted areas revealed altered gene expression profiles compared with those from nonaffected areas.

**Conclusions:**

The agreement between the array data and qRT-PCR analyses validates this multispecies microarray. The microarray measurement of gene expression in zebrafish, which are phylogenetically distant from turbot, indicates that this multispecies microarray will be useful for measuring endocrine responses in other fish.

In 1996 the European Community defined an endocrine disruptor as “an exogenous substance or mixture that alters function(s) of the endocrine system and consequently causes adverse effects in an intact organism or its progeny or (sub)populations” (International Programme on Chemical Safety 2002). Endocrine disruptors of concern include plasticizers such as phthalates and alkylphenols, pesticides, fungicides, detergents, dioxin, polychlorinated biphenyls, and pharmaceuticals such as the synthetic estrogen 17**α**-ethynylestradiol. These xenobiotics are discharged into rivers, lakes, and oceans, where they accumulate in aquatic species. Humans and wildlife are exposed to these compounds directly and through fish and shellfish consumption. In addition, humans are exposed to endocrine disruptors via polluted drinking water.

Some endocrine disruptors interfere with normal endocrine responses because the chemical has structural similarities to hormones such as steroids. As a result, the endocrine disruptors bind to a hormone receptor or to an enzyme that catalyzes hormone synthesis or degradation (Atanasov et al. 2005; Baker 2001). Elevated concentrations of xenobiotics in the environment have raised awareness of their potential impact on human health (Tyler et al. 1998). Exposure of humans to endocrine disruptors may lead to increased rates of fetal death (Bell et al. 2001), intellectual impairment in children (Jacobson and Jacobson 1996), premature puberty in females (Herman-Giddens et al. 1997), and decreased reproductive ability in males (Sharpe and Skakkebaek 1993).

Among endocrine disruptors, alkyl phenol ethoxylates, such as nonylphenol, have been widely studied because of their wide diffusion in the environment through their use in the plastics industry and in detergents, paints, herbicides, and pesticides (Soto et al. 1991). An estimated 60% of man-made alkylphenols enter the aquatic environment (Naylor et al. 1992), with most entering via sewage treatment works, where they are readily degraded to form relatively stable metabolites (Ahel et al. 1987). Nonylphenol is the predominant degradation product of the alkylphenols encountered in the aquatic environment (Giger at al. 1984). Exposures of cell cultures and laboratory animals to nonylphenol have demonstrated that it competes with estradiol for binding to the ER but has only weak estrogenic activity. As a result, there are concerns that exposure of humans and fish to nonylphenol will disrupt male and female reproduction and development.

It also is clear that xenobiotics affect other hormone pathways, such as thyroid hormone (Boas et al. 2006; Zhou et al. 2000). Thus, there is a need for a tool that can screen many endocrine responses in fish taken from polluted water and monitor harmful effluents entering the ecosystem. Ideally suited for this purpose are microarrays, which can simultaneously measure the level of expression of hundreds of genes from a single tissue sample in each animal collected from a polluted environment (Benson and Di Giulio 2008; Hardiman 2004; Hardiman and Carmen 2006; Marton et al. 1998). Microarray analysis of alterations—either up or down—in the levels of genes involved in physiologic responses to estrogens, androgens, glucocorticoids, thyroid hormones, and detoxification of chemicals provides a powerful tool for obtaining a more complete diagnosis of endocrine disruption in fish. A microarray profile of alterations in gene expression associated with a single compound represents a unique signature that can be used to detect compounds with endocrine-disrupting activity in the environment. Moreover, in addition to the practical use of microarrays for analysis of endocrine disruption in fish, microarrays can provide molecular information for elucidating the mechanism of action of nonylphenol, other xenobiotics, and endogenous hormones such as estradiol.

The development of the microarray described here was motivated by the needs of the Los Angeles County Sanitation Districts (LACSD), Orange County Sanitation District (OCSD), the City of Los Angeles Environmental Monitoring Division, the City of San Diego Ocean Monitoring Program, the Southern California Coastal Water Research Project (SCCWRP), and university research groups in Long Beach, Riverside, and San Diego, California (USA). Together, these investigators monitor and study chemical and waste effluents discharged into the coastal marine environment and watersheds through biannual collection of sentinel fish at different sites from San Diego to Santa Barbara to assess accumulated levels and effects of environmental chemicals.

The sentinel fish, which are used to charac terize the presence of chemicals in the marine environment, are hornyhead turbot (*Pleuronichthys verticalis*) and other flatfish, because these fish are often bottom feeders and are at higher risk of exposure to chemicals that accumulate in sediments. These fish also live in a limited area, which allows one to localize the site of chemical pollution. The OCSD has supported several studies that captured turbot from different sites for the analyses of aberrant morphology in organs, for endocrine disruptors in liver tissue and blood, and for vitellogenin, a biomarker for estrogen exposure (Deng et al. 2007; Rempel et al. 2006). Microarray and quantitative reverse-transcriptase polymerase chain reaction (qRT-PCR) analysis of turbot organs would provide enhanced sensitivity to xenobiotics in the marine environment and facilitate control of toxic effluents. Unfortunately, few genes of turbot and other flatfish have been sequenced, which limits the use micro-arrays and qRT-PCR to study disruption of endocrine responses.

In this study, we present data from a multigene cross-species microarray, which we used to analyze gene expression in hornyhead turbot collected in the coastal waters of Orange County and Los Angeles County, California. Parallel experiments with qRT-PCR verified the microarray data. We also used the multi-species microarray to study gene expression in livers of zebrafish (*Danio rerio*) exposed to estradiol and nonylphenol. The use of the multispecies microarray to study gene expression in hornyhead turbot and zebrafish, which are phylogenetically distant (Figure 1), suggests that this array will be a useful diagnostic screening tool to monitor responses to contaminants in Perciformes, Pleuronectiformes, and fish from other taxa for which there are limited genomic sequence data.

## Materials and Methods

### Estradiol and 4-nonylphenol exposure studies in zebrafish

Details of exposure of zebrafish to estradiol and 4-nonylphenol are provided in the Supplemental Material (available online at http://www.ehponline.org/members/2008/11627/suppl.pdf).

### Hornyhead turbot vitellogenin and estradiol assay

Details of the measurements of vitellogenin, estradiol, cortisol, and testosterone (Kelley et al. 2001; Rempel et al. 2006) are provided in the Supplemental Material (available online at http://www.ehponline.org/members/2008/11627/suppl.pdf).

### Environmental hornyhead turbot sample collection

We collected male hornyhead turbot off of the coast of Southern California as part of a Southern California regional marine monitoring study (Bight Field Sampling and Logistics Committee 2003). We used livers from three individual fish from a monitoring station near OCSD outfall and four individuals from a monitoring station near the LACSD outfall for microarray analysis. These fish exhibited morphologic abnormalities, high levels of vitellogenin and estradiol, low levels of cortisol, and histologic abnormalities, such as the presence of immature oocytes (eggs) within the testis (Table 1). We obtained control fish from a monitoring station in Dana Point, California, an area considered relatively nonaffected, and maintained them in a clean-water laboratory setting for 4 weeks. The vitellogenin level in the control male turbot measured with an enzyme-linked immunosorbent assay (ELISA) was 0.0037 ng/μg protein, 27- to 700-fold lower than the vitellogenin levels in the turbot collected from the polluted OCSD and LACSD sites (Table 1). This indicates that the control male turbot were not exposed to an estrogenic compound, which validates the use of their liver RNA as a control for the microarray and the qRT-PCR analyses.

Exposed animals were sacrificed immediately after capture; the livers were harvested and frozen in liquid nitrogen and stored at −70°C. All the animals were treated humanely and with regard for alleviation of suffering.

### Construction of the multispecies micro array

**Figure 1:**
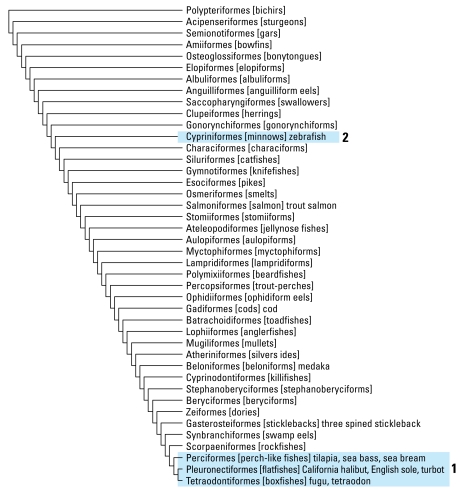
Flatfish (Pleuronectiformes) in an evolutionary context. Adapted from the phylogeny (Coleman 2004; Helfman et al. 1997). Tetraodontiformes (*Fugu*, *Tetraodon*) and Perciformes (cichlid, tilapia, sea bass, sea bream, perch) are close phylogenetic relatives of Pleuronectiformes (turbot, halibut, sole) (box 1). Cypriniformes (zebrafish) are distant phylogenetic relatives (box 2).

To overcome the scarcity of sequence data in GenBank (National Center for Biotechnology Information 2008) for hornyhead turbot, we constructed a 65mer oligonucleotide-based microarray containing conserved sequences from genes of interest. We designed oligo probes by collecting available fish sequences in GenBank for a given gene (e.g., *ESR1/ Er***α**, *Vtg*, *CYP3A*, and *FXR*) using BLAST (Altschul et al. 1990). We selected sequences from Tetraodoniformes (*Fugu*, *Tetraodon*) and Perciformes (cichlid, tilapia, sea bass, sea bream), which are close, from a phylogenetic perspective, to Pleuronectiformes (hornyhead turbot, California halibut) (Figure 1). We also used available sequences from medaka, stickleback, and zebrafish, in addition to some hornyhead turbot–specific cDNA sequences obtained by degenerate PCR cloning. To design 65mer microarray probes, we used the Clustal X program to construct multiple alignments to uncover conserved regions (Thompson et al. 1997), identified nucleotide sequences within 1,200 bases from the mRNA 3′ end, and used the OligoWiz program to analyze them (Nielsen et al. 2003; Wernersson and Nielsen 2005). We subjected each copy of an individual gene in several fish to a pairwise BLAST comparison with the corresponding gene from other fish to ensure that the DNA sequence was between 80% and 90% identical, thereby increasing the likelihood that the homologous turbot sequence would contain at least 85% identity to one of the oligonucleotides. Gene names and corresponding accession numbers are in Supplemental Material, Table 1 (available online at http://www.ehponline.org/members/2008/11627/suppl.pdf).

Oligonucleotides were synthesized to contain a 5′ amine group and desalted by Operon Technologies (Alameda, CA) and Invitrogen (Carlsbad, CA), and used without further purification. Oligonucleotides were printed on amine-reactive silanized glass slides (Surmodics, Inc., Eden Prairie, MN) at the University of California, San Diego BioMedical Genomics Microarray Facility as described previously (Hardiman et al. 2003).

### RNA extraction, fluorescent target labeling, and microarray hybridizations

We converted 500 ng of total RNA into fluorescently labeled cyanine 3 (Cy 3) or 5 (Cy 5) cRNA using the Low RNA Input Fluorescent Linear Amplification Kit (Agilent Technologies, Santa Clara, CA). Fluorescent targets were purified to remove unincorporated nucleotides using RNeasy (Qiagen, Carlsbad, CA). We used absorbance (optical density) at 260 nm to quantify the cRNA concentrations, and absorbance at 550 nm and 650 nm to measure the efficiency of Cy 3 and Cy 5 dye incorporation. Hybridization was carried out for 18 hr at 42°C in a shaking incubator at 100 rpm. The microarrays were washed with 1× saline–sodium citrate (SSC)/0.2% sodium dodecyl sulfate (SDS) for 5 min at room temperature followed by two 5-min washes with 0.1× SSC/0.2% SDS at room temperature. The microarray was rinsed briefly with water and dried by centrifugation at 800 rpm for 5 min. Technical replicate hybridizations were carried out with each sample. Slides were scanned using an Axon 4000A scanner (Molecular Devices, Sunnyvale, CA) at the photomultiplier tube settings of 500 V for Cy 3 and 600 V for Cy 5.

### Amplification and sequencing of horny-head turbot mRNAs

We amplified partial turbot transcripts using conserved sequences from other fish species to guide the choice of primer design. Gene-specific primers were designed using Primer3 software (Rozen and Skaletsky 2000) as outlined in the Supplemental Material (available online at http://www.ehponline.org/members/2008/11627/suppl.pdf). All the amplicons were directly sequenced using the respective forward and reverse PCR primers. We subjected all of the sequencing reads to a series of quality control measures, including a Phred quality score > 20 from the Phred program (Ewing et al. 1998), and manual trace inspection. The identity of each sequence was confirmed by performing BLAST searches of GenBank.

### Microarray data analysis

Array data has been deposited in the ArrayExpress Database (accession numbers E-MTAB-43 and E-MTAB-44) (European Bioinformatics Institute 2008). Statistical analysis of the microarray experiment involved two steps: normalization of microarray data and sorting of the genes according to interest. We normalized all samples simultaneously using a multiple-loess technique described previously (Šášik et al. 2004). In designing the interest statistic, we borrowed ideas from the software package Focus (Cole et al. 2003). The interest statistic reflects a biologist’s understanding that a gene with a greater fold change (in absolute value) than other genes is potentially more interesting. Also, given two genes with the same fold changes, the gene with a higher expression level (and therefore higher absolute change) is more relevant. Ogawa et al. (2004) described this approach in greater detail.

### Differential expression and signal intensity measurements

To investigate alterations in gene expression of controls and exposed fish, we used two independent analytical methods, MA plots (M, the intensity ratio, versus A, the average intensity for a dot in the plot) and normal quantile (q-q) plots, as described in detail in Supplemental Material (available online at http://www.ehponline.org/members/2008/11627/suppl.pdf).

### qRT-PCR analysis

We measured relative turbot mRNA transcript levels by qRT-PCR in a LightCycler 480 (Roche Applied Science, Indianapolis, IN). We extracted total RNA from hepatic turbot samples as described above, reverse-transcribed it using the Roche Transcriptor kit, and quantified 50 ng cDNA using the LightCycler 480 SYBR Green Master kit (Roche Applied Science). The 18S rRNA served as an internal control for normalization. Each sample was run in triplicate and mean values were reported. Normalized gene expression values were obtained using LightCycler Relative Quantification software (Roche Applied Science). Relative gene copy numbers were derived using the formula 2**^Δ^**^CT^, where **Δ**CT is the difference in amplification cycles required to detect amplification product from equal starting concentrations of turbot liver RNA.

## Results

### Design of the multispecies endocrine microarray

Development of the multi species microarray was motivated by the mission of the participating sanitation districts, university research groups, and SCCWRP to monitor the endocrine status of turbot, halibut, and other flatfish at different sites in coastal Southern California. We wanted a broad measure of the effects of chemicals on a variety of endocrine responses in fish. Thus, the microarray included probes for genes encoding receptors for estradiol (*ESR1/ Er***α**; *ESR2/Er***β**), progesterone (*PR*), testosterone (*AR*), cortisol (*GR*), aldosterone (*MR*), thyroid hormone (*THRA/TR***α**; *THRB/TR***β**), retinoids (*RAR*, *RXR*), and vitamin D (*VDR*), as well as other nuclear receptors: farnesoid X receptor (*FXR*), pregnane X receptor (*PXR*), hydroxysteroid dehydrogenases, and detoxification enzymes (*CYP1A1*, *CYP3A*) [see Supplemental Material, Table 1 (available online at http://www.ehponline.org/members/2008/11627/suppl.pdf)]. This provided a diagnostic tool for measuring altered expression of genes that are important in several endocrine pathways in fish, which increased the scope of detection for the presence of endocrine disruptors in coastal waters off Southern California.

**Figure 2:**
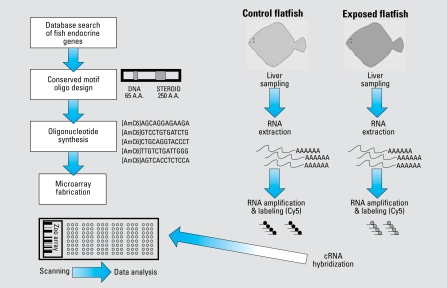
Schematic representation of the design and application of a multispecies microarray-based test to monitor xenoestrogen exposure for environmental monitoring.

A challenge in developing this platform was the paucity of available sequence information for hornyhead turbot in GenBank. To overcome this problem, we searched GenBank for sequences in other fish to find regions of sequence conservation that could be used to construct a microarray slide that could detect altered gene expression in multiple fish species. Fortunately, the genomes of two genera of Tetraodontiformes, *Fugu* and *Tetraodon*, have been sequenced. Moreover, many genes from various Perciformes genera were in GenBank. Tetraodontiformes and Perciformes are phylogenetically close to Pleuronectiformes, as shown in Figure 1. Also of importance was the extensive catalog of sequenced genes from zebrafish, which is distant from of Pleuronectiformes. Sequences conserved in zebrafish, *Fugu*, *Tetraodon*, and various Perciformes were likely to be conserved in turbot. A schematic representation of the design and application of the multispecies microarray test to monitor xenobiotic exposure is presented in Figure 2.

### Differential expression and signal intensity measurements using the multispecies micro-array

**Figure 3:**
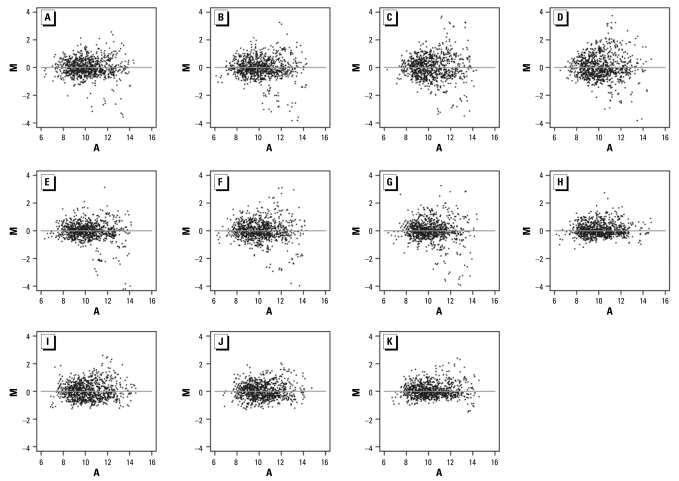
MA plots of differential expression and signal intensity measurements using the multispecies endocrine microarray for control and exposed fish (loess normalization). (*A–D*) Individual LACSD-exposed fish versus pooled controls. (*E–G*) Individual OCSD-exposed fish versus pooled controls. (*H*) Pooled controls versus pooled controls. (*I–K*) Individual control fish versus pooled control. Each point represents data from a single 65mer oligonucleotide probe. M is a measure of differential gene expression [log_2_ (exposed /control)] in *A–G* or absence of significant differential gene expression in the self–self plots [log_2_ (control/control intensity)] in *H*–*K*. A is a measure of signal intensity [(0.5 log_2_ exposed intensity + 0.5 log_2_ control intensity) in *A–G* or (0.5 log_2_ control intensity + 0.5 log_2_ control intensity) in *H–K*].

We assessed male hornyhead turbots sampled at OCSD and LACSD for exposure to xenoestrogens using the multispecies microarray. Figure 3 shows the measurements obtained using the multispecies microarray to examine control and exposed fish. We determined alterations in gene expression in horny-head turbot liver relative to control fish by using a threshold of log_2_ intensity ratio ≥ 2. The MA plots revealed differential gene expression profiles between exposed and control turbot taken from sites in LACSD (Figure 3A–D) and OCSD (Figure 3E–G), and an absence of significant differential gene expression in the control self–self plots (Figure H–K).

**Figure 4:**
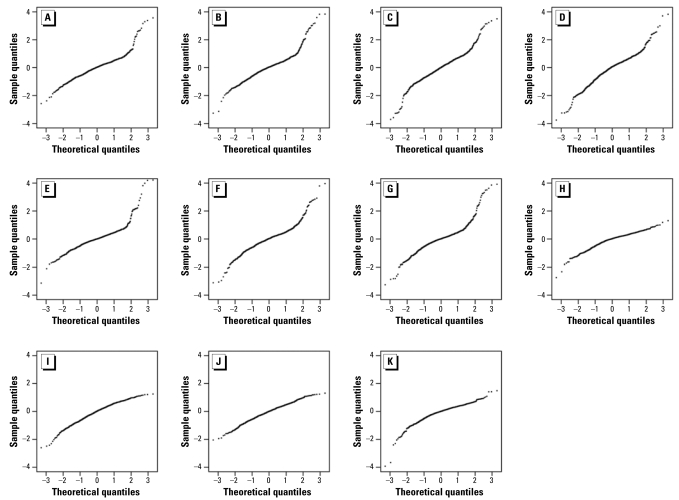
Normal q-q plots of multispecies endocrine microarray data. The q-q plots were constructed to determine whether control and exposed fish data sets derived from populations have a common distribution. (*A–D*) Individual LACSD-exposed fish versus pooled controls. (*E–G*) Individual OCSD-exposed fish versus pooled controls. (*H*) Pooled controls versus pooled controls. (*I–K*) individual control fish versus pooled control. The q-q plots show the distribution of the log_2_ (exposed/control) fold changes and the deviation, if any, from a normal Gaussian distribution. When the two data sets derive from a population with the same distribution, the points fall approximately along this straight line, as is the case with the control sample data populations, both pooled and individual (*I–K*). When the two data sets derive from populations with different distributions, the data deviate from this straight line (*A–G*). Exposed samples differ from the control, with a sharp rise observed in the quantile curve at log_2_ ratios of 2, indicating the presence of large log_2_ ratios and true differences in gene expression.

We employed q-q plots to examine more closely the differences in expression between the control and exposed fish. The q-q plots in Figure 4 demonstrate the distribution of the log_2_ (exposed/control) fold changes and the deviation from a normal Gaussian distribution. When a data set derives from the Gaussian distribution, the normal-quantile plot is a straight line. The plots in Figure 4H–K show that the observed log_2_ ratio between control fish, both pooled and individual, is reasonably close to a Gaussian. This distribution is due to individual variation in fish combined with unavoidable random experimental errors.

The log_2_ ratio between exposed and control fish (Figure 4A–G) results in curved ends of the q-q line, which indicate the presence of heavy tails in the distribution of log_2_ (exposed/control). The exposed samples clearly differ from the control samples. Specifically, the sharp increase in the quantile curve at a log_2_ ratio of about 2 suggests that genes with |log_2_ (exposed/control)| > 2 show significant regulation in the LACSD-exposed (Figure 4A–D) and OCSD-exposed (Figure 4E–G) fish compared with controls, which is in agreement with the MA plots in Figure 3.

### Gene expression patterns in male turbot from Southern California coastal regions

**Figure 5:**
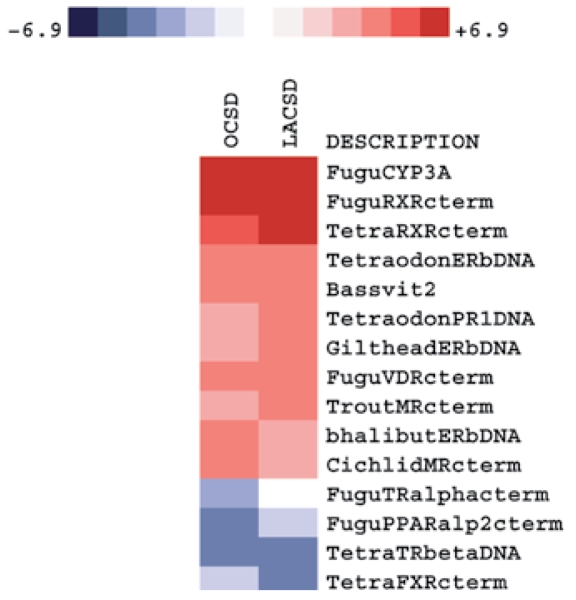
Gene expression profiling of male turbot liver collected at OCSD and LACSD (contaminated) and control fish from a nonaffected area. This heat map depicts fold changes observed between exposed and control fish. LACSD and OCSD data derived from four and three independent biologic replicate microarray experiments, respectively.

A heat map of selected genes that were either strongly down-regulated or up-regulated in fish collected near the OCSD and LACSD outfalls relative to controls is presented in Figure 5. Fish sampled at both affected sites exhibited strong increases in the expression of *CYP3A*, *RXR*, *ER***β**, *Vtg2*, and *MR* relative to control fish. The *VDR* was also up-regulated compared with control fish. Transcripts encoding *FXR* and peroxisome proliferator-activated receptor **α** (*PPAR***α**) were down-regulated compared with control fish. Additionally, thyroid receptor **α** and **β** mRNAs were down-regulated.

### qRT-PCR analysis of turbot gene expression

**Figure 6:**
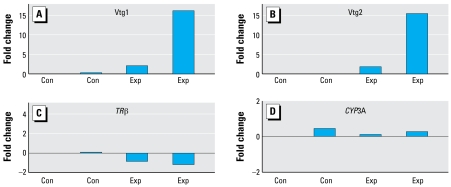
Multispecies SYBR Green qRT-PCR validation of multispecies endocrine microarray for *Vtg1* (*A*), *Vtg2*( *B*), thyroid hormone receptor **β** (*C*), and *CYP3A*-specific transcripts (*D*) in livers from control (Con) and exposed (Exp) hornyhead turbot (each bar represents one fish). 18S rRNA served as an internal control for normalization. Plots are mean fold changes from triplicate measurements, relative to control fish. *Vtg1* (*A*) and *Vtg2* (*B*) transcripts were strongly up-regulated (> 15 fold) in one exposed fish. *TR***β** was down-regulated in two control fish. *CYP3A* was up-regulated in three fish and down-regulated in one fish.

In order to use qRT-PCR to determine whether the microarray data were accurately monitoring changes in hepatic gene expression in the turbot, we cloned, via RT-PCR, partial fragments corresponding to highly conserved regions in the 28S rRNA, *CYP3A*, *TR***β**, vitellogenin 1 (*Vtg1*), and *Vtg2*. We confirmed identities of the fragments by DNA sequencing, and sequence data have been deposited in GenBank (accession numbers FJ042791–FJ042800). We used these short turbot-specific sequences for SYBR Green quantitative PCR experiments on individual turbot from affected regions. As shown in Figure 6, we observed greatest differences with the *Vtg1* and *Vtg2* transcripts, with > 15-fold induction in one turbot. *TR***β** was down-regulated in two control fish examined. *CYP3A* was up-regulated in three fish. Thus, these qRT-PCR data validate the microarray analysis for these genes.

Moreover, in control turbot, Vtg1 was 0.0037 ng/μg protein, as determined by an ELISA assay. Thus, vitellogenin levels in control male turbot were 27- to 700-fold lower than those found in turbot collected from polluted sites in Orange County and Los Angeles County (Table 1). The agreement between vitellogenin measured with the ELISA assay and with the qRT-PCR analysis (Figure 6) gives us confidence in qRT-PCR analyses of other genes in male turbot taken from polluted sites.

### Gene expression patterns in zebrafish exposed to estradiol and 4-nonylphenol

We investigated alterations in gene expression in zebrafish liver using the multispecies micro-array after exposing fish for 2 weeks to either the xenoestrogen 4-nonylphenol or estradiol at 10^−7^ M. We carried out these experiments to determine if probes designed from conserved sequences from Tetraodoniformes and Perciformes were able to hybridize to genes in zebrafish (Cypriniformes), which are distant phylogenetic relatives of Pleuronectiformes (Figure 1).

**Figure 7:**
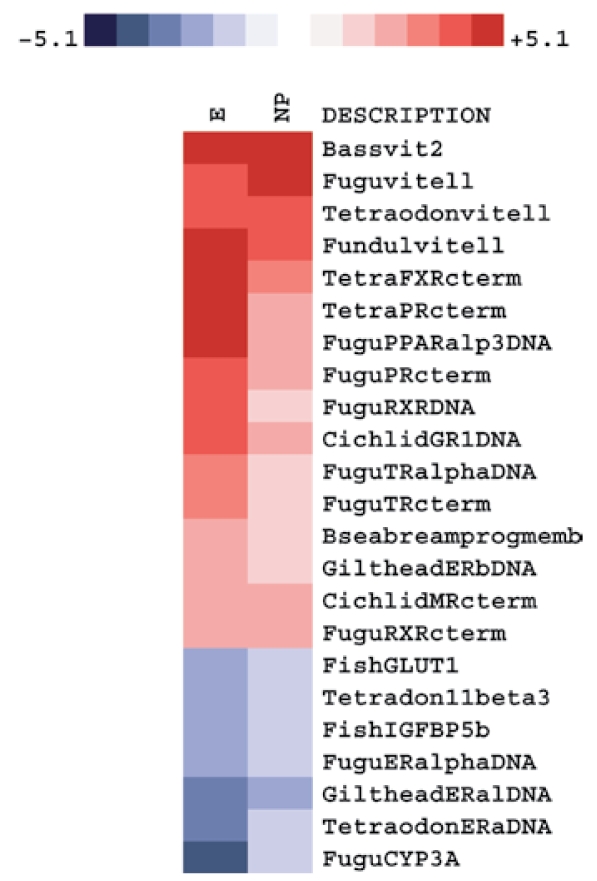
Cross-species applicability of the Multispecies endocrine microarray: detection of alterations in gene expression in zebrafish liver after a 2-week exposure to either 4-nonylphenol or estradiol. This heat map depicts fold changes between exposed and control fish. Data derived from four independent biological replicate experiments.

The data in the heat map in Figure 7 show that estradiol produced a broader and higher response than did 4-nonylphenol. Both treatments induced strong up-regulation of vitellogenin, several nuclear receptors, proteins involved in oogenesis, and steroid metabolism. Among these, the *FXR* transcript was the most up-regulated. Other transcripts that were strongly up-regulated included *PR*, *MR*, *PPAR***α**, *PXR*, retinoid X receptor (*RXR***α**), *TR***α**, and *GR*.

Other transcripts including *ER***α**, the glucose transporter *GLUT*, an insulin-like growth factor binding protein (*IGFBP*) (Kelley et al. 2002), and 11**β**-hydroxysteroid dehydrogenase type 3 (*11***β***HD3*) (Baker 2004) were down-regulated by treatment with estradiol and 4-nonylphenol. Estradiol also induced a strong decrease in both *CYP3A* and *ER***α** expression. Although *CYP3A* expression was down-regulated in 4-nonylphenol–treated fish, the response was muted compared with estradiol.

## Discussion

There is an increasing demand for robust bio-assays that provide a comprehensive assessment of the effects of chemical contaminants in aquatic populations. Microarrays are ideally suited for this purpose because they can detect changes in many genes in a single tissue sample, providing snapshots of alterations in endocrine pathways in normal and contaminated fish. This knowledge identifies gene families and biochemical pathways that are affected, in addition to identifying those that remain unaffected (Vilo and Kivinen 2001). A challenge in developing a microarray platform to study alterations in gene transcription in sentinel coastal species was the paucity of genomic sequence data. To overcome this obstacle, we constructed a 65mer oligonucleotide-based microarray containing conserved sequences from genes of interest. The novelty of this platform is that it used highly conserved probes from several fish species, permitting application of the array to studies involving turbot and zebrafish. We used 65mers to accommodate sequence differences, polymorphic regions, and species-specific codon use. We reasoned that this approach would be successful because oligonucleotides 50 nucleotides in length have been previously demonstrated to hybridize to RNA sequences that differ by 15% in their overall sequence (Li et al. 2005; Nielsen et al. 2003). We focused the array on key targets with defined roles in endocrine pathways and processes, in addition to bio-markers for contaminant exposure.

We used the multispecies microarray to assess alterations in hepatic gene expression in male hornyhead turbots that we collected during a regional marine monitoring study at two monitoring stations in the Southern California Bight 2003 Survey that are considered affected by pollution (Bight Field Sampling and Logistics Committee 2003). The assessed fish had been previously examined for exposure to xenoestrogens using the classical biomarker vitellogenin, measurements of plasma levels of testosterone and estradiol, and anomalies in gonadal morphology (Deng et al. 2007; Rempel et al. 2006).

### Microarray analysis of turbot exposed to pollutants

Microarray analysis detected differences in hepatic gene expression patterns in exposed turbot from the two monitoring areas compared with control individuals. Exposed turbot showed up-regulation of *CYP3A* and *RXR*. Interestingly, thyroid receptors **α** and **β** were down-regulated in fish from both monitoring areas, indicating the presence of compounds that are able to interfere with the thyroid response.

Another example of the utility of micro-array analysis of fish from polluted areas is a study comparing transcripts in male flounder collected from an affected site (Tyne) and a reference site (Alde) in the United Kingdom (Williams et al. 2003). Eleven transcripts were differentially expressed between the two groups: in the Tyne male fish, seven transcripts were more highly expressed [*CYP1A1*, *UDPGT* (UDP-glucuronosyltransferase), **α**-2HS-glycoprotein, dihydropyrimidine dehydrogenase, Cu/Zn superoxide dismutase, aldehyde dehydrogenase, and paraoxonase], whereas four transcripts [elongation factor 1 (*EF1*), *EF2*, *Int-6* (translation initiation factor 3 subunit 6), and complement component C3 mRNA] were statistically significantly less abundant (Williams et al. 2003).

### Microarray analysis of zebrafish exposed to estradiol and 4-nonylphenol

We observed differences between estradiol and 4-nonylphenol in the strength of the estrogen-like response, which we attribute to 4-nonylphenol having a low affinity for the ER (Kuiper et al. 1997). The data on gene expression in zebrafish provide another validation of the multispecies microarray and demonstrate its potential for investigating gene expression in Pleuronectiformes and Perciformes.

We found up-regulation of vitellogenin, *PR*, *RXR*, and *ER***β** transcripts in male zebrafish exposed to both estradiol and 4-nonyl phenol. Up-regulation of vitellogenin and *PR* are well-established responses to estrogens. *RXR* has been shown to be up-regulated by the estrogenic chemical bisphenol A in murine embryos (Nishizawa et al. 2005). *ER***β** expression has been shown to increase upon exposure to xenoestrogens in zebrafish (Islinger et al. 2003) and to 4-nonylphenol in many fish species (Soverchia et al. 2006). Up-regulation of *ER***β** was reported after exposure to alkylphenols in juvenile goldfish, medaka, rainbow trout, and zebrafish (Inui et al. 2003; Islinger et al. 2003; Soverchia et al. 2006; Vetillard and Bailhache 2006), indicating that it can be considered a biomarker for xenoestrogen exposure.

*ER***α** express ion was repressed with both estradiol and 4-nonylphenol treatments. Distinct patterns of expression for *ER***α** and *ER***β** have been documented in fish (Choi and Habibi 2003), but differences in the interaction of xenoestrogens with the two subtypes of estrogen receptors have not been well characterized.

After exposure to 4-nonylphenol, we observed a modest repression of *CYP3A* in zebrafish. However, we observed a strong repression of *CYP3A* mRNA after estradiol exposure. Similar results have been reported in trout (Pajor et al. 1990), suggesting an important role of the sex hormones in *CYP3A* expression, which is further supported by the sexually dimorphic expression of *CYP3A* genes reported in many fish species (Hasselberg et al. 2004; Hegelund and Celander 2003).

Interestingly, expression of the *PXR*, a sensor for xenobiotics (Moreau et al. 2008; Xie et al. 2000), was induced by both compounds. PXR mediates the effects of 4-nonylphenol on the activation of *CYP3A* genes in mouse, rat, and humans (Masuyama et al. 2000, 2002). In juvenile Atlantic salmon, increases in *PXR* and *CYP3A* transcript levels have been observed after 4-nonylphenol exposure, suggesting a mechanism similar to that reported in mammalian systems (Meucci and Arukwe 2006). Furthermore, hepatic expression of *CYP3A* is induced by the organochlorine pesticide methoxychlor in male largemouth bass, *Micropterus salmoides* (Blum et al. 2008).

We also found strong activation of *FXR*, *PPAR*, and *RXR***α** expression in zebrafish exposed to estradiol or 4-nonylphenol, which indicates that xenoestrogens can affect a variety of physiologic pathways. PPAR is involved in the regulation of lipid-metabolism–related genes, and its interaction with xenobiotic compounds is thought to be responsible for alterations in adipogenesis and diseases such as obesity in humans (Grun and Blumberg 2006). FXR is strongly activated by bile acids and serves as a central coordinator for bile acid biosynthesis metabolism and transport. Possible interaction of xenobiotics with FXR could lead to changes in bile acid homeostasis and hepatic toxicity. Our finding that *FXR* was up-regulated in zebrafish exposed to estradiol and 4-nonylphenol has not been reported previously in any fish. This finding demonstrates the utility of microarrays in uncovering the effects of hormones and chemicals, which can subsequently be used to construct a profile for exposure to a given chemical.

Of practical importance for the analysis of zebrafish exposed to estradiol and 4-nonyl-phenol is that the zebrafish belongs to the order Cypriniformes and is phylogenetically distant from Tetraodoniformes, Perciformes, and Pleuronectiformes, whose sequence information was used to guide the design of the array probes. Thus, the data obtained from the zebrafish experiments indicated that the multi-species microarray possesses cross-species utility.

## Conclusions

The results we obtained using the multispecies microarray to assess male hornyhead turbots in two coastal areas considered affected by pollution revealed the presence of a mixture of endocrine disruptors containing xenoestrogens, and most likely xenobiotics capable of inter acting with the thyroid system. These results highlight the utility of the multispecies microarray as a diagnostic for the presence of endocrine disruptors in the aquatic environment. We demonstrated the broad use of the multispecies microarray to study the effects of environmental chemicals on fish in its application to zebrafish exposed to chemicals in a laboratory and a sentinel species (hornyhead turbot, *Pleuronichthys verticalis*) collected from polluted sites. The results presented here demonstrate the feasibility of adding other genes of interest in fish physiology to a second-generation multispecies microarray for characterizing fish exposed to pollutants.

## Correction

Figure 7 was incorrect in the original article published online. It has been corrected here.

## Figures and Tables

**Table 1:** Characteristics of hornyhead turbots sampled.

Location	Station ID	Sample ID	Sex	Cortisol (ng/mL)	Estradiol (pg/mL)	IGF (ng/mL)	Thyroxine (ng/mL)	Vitellogenin (ng/μg protein)	Morphology diagnosis	Lesion grade	Maturity stage
OCSD	4041	3	M	8.0	58.1^a^	16.0	0.7	0.1	Oocytes^a^	Minimal	Stage 1
OCSD	4041	4	M	18.6	134.0^a^	20.2^a^	2.5^a^	0.2	Oocytes, macrophage aggregates^a^	Minimal	Stage 1
OCSD	4041	6	M	89.4^a^	90.8^a^	17.1	0.5	0.4	None	NA	Stage 1
LACSD	4086	1	M	20.7^a^	2.2	16.3	0.4	1.5^a^	Fibrous septa	Minimal	Stage 1
LACSD	4086	2	M	15.1	2.2	17.0	0.4	2.6^a^	Fibrous septa	Moderate	Stage 2
LACSD	4086	3	M	1.1	21.3	15.9	0.4	1.0	None	NA	Stage 2
LACSD	4086	4	M	8.4	2.2	17.4	0.5	1.6	Oocytes, fibrous septa^a^	Moderate	Stage 1

Abbreviations: IGF, insulin-like growth factor; M, male; NA, not applicable. Individuals with morphologic abnormalities induced by endocrine disruptors were chosen for microarray experiments. Morphologic lesions (not caused during capture) and maturity stages are noted.

a Anomalies.
